# Serine 207 phosphorylated lysyl-tRNA synthetase predicts disease-free survival of non-small-cell lung carcinoma

**DOI:** 10.18632/oncotarget.18053

**Published:** 2017-05-22

**Authors:** Suliman Boulos, Min Chul Park, Marian Zeibak, Shen Yun Foo, Yoon Kyung Jeon, Young Tae Kim, Alex Motzik, Sagi Tshori, Tamar Hamburger, Sunghoon Kim, Hovav Nechushtan, Ehud Razin

**Affiliations:** ^1^ Department of Oncology, Hadassah Hebrew University Hospital, Jerusalem, Israel; ^2^ Medicinal Bioconvergence Research Center, Seoul National University, Seoul, Korea; ^3^ Department of Biochemistry and Molecular Biology, Institute for Medical Research Israel-Canada, The Hebrew University of Jerusalem, Jerusalem, Israel; ^4^ NUS-HUJ-CREATE Cellular & Molecular Mechanisms of Inflammation Program, Department of Microbiology and Immunology, National University of Singapore, Singapore; ^5^ Department of Pathology, Seoul National University Hospital, Seoul, Korea; ^6^ Department of Thoracic and Cardiovascular Surgery, Seoul National University Hospital, Seoul, Korea; ^7^ Kaplan Medical Center, Rehovot, Israel; ^8^ Sharett Institute of Oncology, Hadassah Hebrew University Hospital, Jerusalem, Israel

**Keywords:** LysRS, P-s207 LysRS, multi-synthetase complex, EGFR, non-small-cell lung cancer

## Abstract

It has been shown that various tRNA synthetases exhibit non-canonical activities unrelated to their original role in translation. We have previously described a signal transduction pathway in which serine 207 phosphorylated lysyl-tRNA synthetase (P-s207 LysRS) is released from the cytoplasmic multi-tRNA synthetase complex (MSC) into the nucleus, where it activates the transcription factor MITF in stimulated cultured mast cells and cardiomyocytes. Here we describe a similar transformation of LysRS due to EGFR signaling activation in human lung cancer. Our data shows that activation of the EGFR results in phosphorylation of LysRS at position serine 207, its release from the MSC and translocation to the nucleus. We then generated a P-s207 LysRS rabbit polyclonalantibody and tested 242 tissue micro-array samples derived from non-small-cell lung cancer patients. Highly positive nuclear staining for P-s207 LysRS was noted in patients with EGFR mutations as compared to WT EGFR patients and was associated with improved mean disease-free survival (DFS). In addition, patients with mutated EGFR and negative lymph node metastases had better DFS when P-s207 LysRS was present in the nucleus. The data presented strongly suggests functional and prognostic significance of P-s207 LysRS in non-small-cell lung cancer.

## INTRODUCTION

Lysyl-tRNA synthetase (LysRS) is an enzyme which catalyzes the ligation of lysine to its cognate transfer RNA (tRNA) during protein synthesis. It belongs to a family of 20 enzymes known as aminoacyl-tRNA synthetases (AARSs). The primary function of this group of enzymes is the aminoacylation of tRNAs. LysRS is part of the intracellular multi-tRNA synthetase complex (MSC) that consists of three non-enzymatic proteins and 8 AARSs [1]. Through evolution, numerous aminoacyl-tRNA synthetase (AARS) have obtained a non-canonical role in signal transduction in both normal and cancerous tissues [2]. In many instances the alternative role was a result either of a post translational modification, different localization or fragmentation of the original molecule.

In some cases, studies on mechanistic aspects of the activation of the non-canonical function of these proteins revealed nuclear translocation [3]. An important non-canonical function of LysRS in mast cells has been previously reported by our group, where LysRS is released from the MSC upon immunological challenge in a MAPK/ERK-dependent manner, undergoes phosphorylation on Serine 207 (P-s207), and translocates to the nucleus [4]. This specific phosphorylation results in LysRS losing its ability to acetylate tRNA but enhances its production of the second messenger diadenosine tetraphosphate (Ap4A). Ap4A has been shown to bind to the tumor suppressor Hint-1, releasing its inhibition on microphthalmia-associated transcription factor (MITF) [5].

Since LysRS is found in all tissues, we postulated that it may be activated not only by immunologic stimulation, but also by other stimuli in nonimmune cells. The present work is focused on the activation of LysRS by Epidermal Growth Factor Receptor (EGFR), as increased EGFR activity has been associated with various types of cancers. In the most deadly cancer type – lung cancer, mutated EGFR was revealed as a major tumor driver [6].

EGFR mutations are present in around 7% of lung adenocarcinoma cases in the western world, and in 30% of lung cancer cases in the Far East [7]. Moreover, activation of wild type EGFR has been demonstrated to have a role in squamous cell lung cancer [8].

As the implications of P-s207 LysRS in human cancers have not been established, we focused our initial studies on lung cancer due to the importance of the EGFR in the prognosis and treatment of this cancer group [6, 8–12]. The signal transduction initiated by EGFR activation results in the downstream phosphorylation of ERK [13–16]. As we reported, mentioned, the MAPK-ERK pathway leads to the phosphorylation of LysRS and its release from the MSC in activated mast cells [4].

To investigate whether EGFR signaling affects LysRS release from the MSC, we developed a polyclonal rabbit antibody specific for P-s207 LysRS. This allowed us to study the presence of P-s207 LysRS in immunohistochemical studies of resected lung cancer tissues, and relate the localization of P-s207 LysRS to cancer prognosis in different subsets of lung cancer patients.

In this study, we confirm the relationship between EGFR activation and LysRS s207 phosphorylation, and its role as a prognostic factor in lung cancer after primary surgery.

## RESULTS

### EGFR triggered release of P-s207 LysRS from the MSC and translocation of P-s207 LysRS to the nucleus in lung cancer

**Figure 1:**
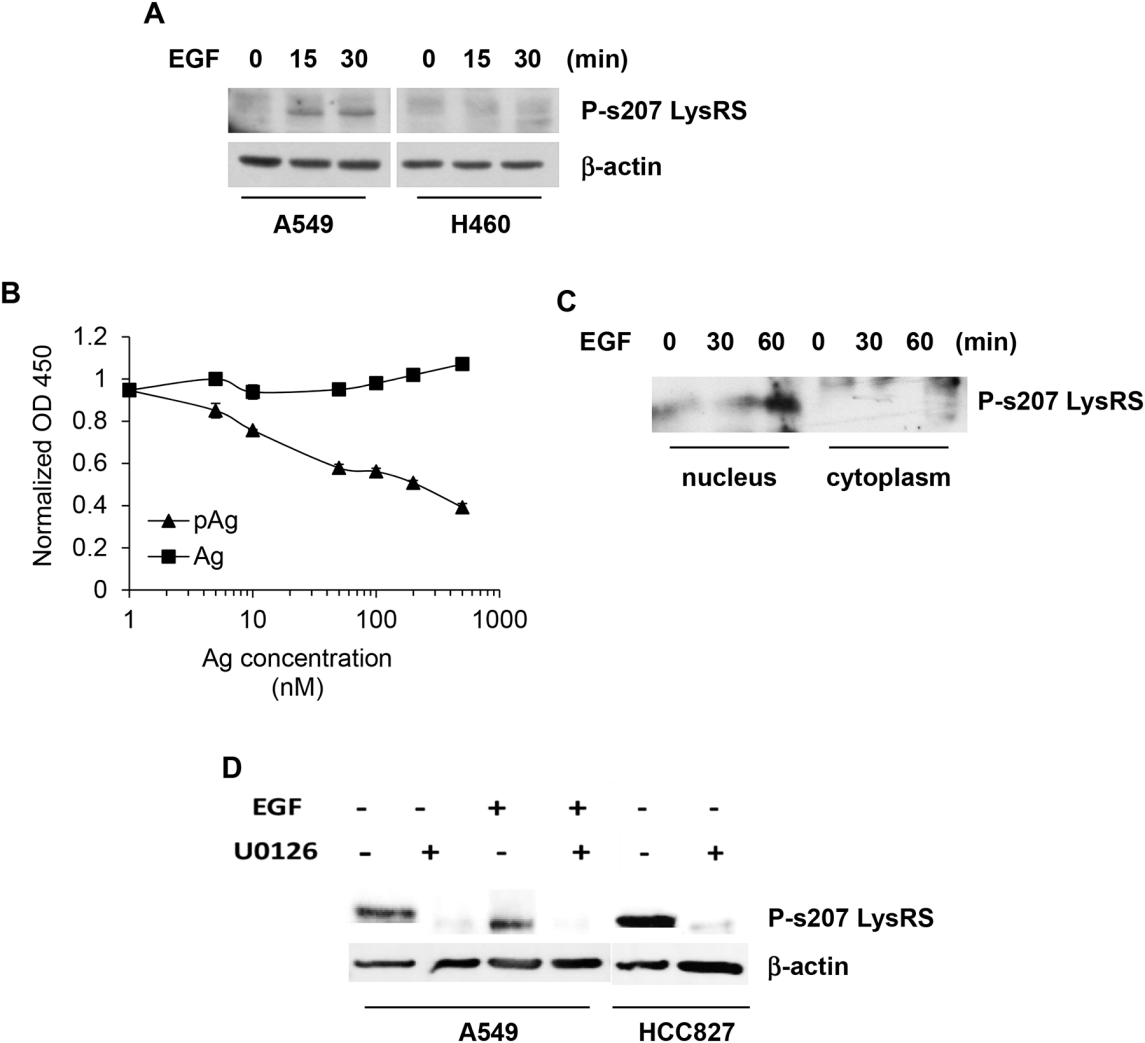
EGFR-dependent LysRS release from the multi-synthetase complex (MSC) and translocation to the nucleus in NSCLC **(A)** A549 cells were treated with 100 ng/ml of EGF for the indicated times. Phosphorylation of P-s207 LysRS was determined by immunoblotting using the rabbit polyclonal anti 207pLysRS antibody **(B)** Competitive ELISA with P-s207 LysRS antibody. P-s207 and wild type LysRS antigens (the peptides EITLLS (p) PCLHM and EITLLSPCLHM, respectively) were used at various concentrations as soluble competitors for P-s207 LysRS **(C)** Nuclear fractionation assay to determine the nuclear levels of P-s207 LysRS in A549 cells. To determine the localization of P-s207 LysRS, A549 cells were starved for 2 hr and incubated with EGF before nuclear and cytoplasmic fractionation. P-s207 LysRS levels were determined by immunoblotting. **(D)** Inhibition of ERK Phosphorylation by U0126 treatment in A549 and HCC827 cell lines. A549 cells were starved for 2hr and treated with EFG (100 ng/ml) for 15 min before exposure to U0126 (10 μM) for 10 min. HCC827 cells were treated with U0126 only. Cells were lysed and levels of P-s207 LysRS were determined by western blotting.

The human EGF trigger of NSCLC cell line, A549, which expresses high levels of EGFR, EGF-sensitivity and lack of EGFR mutations, caused s207 phosphorylation of LysRS (Figure 1A). This as opposed to the EGF-insensitive cell line, H460, which is a large cell lung cancer. The specificity of a new polyclonal antibody targeting P-s207 LysRS was validated by an ELISA competitive assay using P-s207 LysRS and LysRS peptides (Figure 1B). In order to confirm this result, we repeated the ELISA competitive assay using two different phosphorylated peptides, one was a LysRS peptide with a phosphorylation in position T52 (LysRS T52P) and the second peptide was the ribosomal protein S6 (RPS6) phosphorylated in position S240. Both peptides did not interfere with the binding of our P-s207 LysRS antibody.

As P-s207 LysRS has been shown to translocate to the nucleus [4], a nuclear/cytoplasmic fractionation assay was carried out following EGF activation of EGFR. A gradual increase in the nuclear levels P-s207 LysRS were observed (Figure 1C), the experiment was reliable for YY-1, a protein that is found only in the nucleus, and HSP90, that is present only in the cytoplasm (see Supplementary Figure 1). In order to determine whether EGFR/ERK pathway is required for phosphorylation of LysRS, A549 cells were exposed to the ERK inhibitor U0126 prior to their exposure to EGF (Figure 1D). The human EGFR mutated NSCLC cell line, HCC827, were only treated with U0126, as it has constitutively active EGFR. A significant decreased in LysRS phosphorylation was observed in both cell lines treated with U0126 (Figure 1D).

### Nuclear P-s207 LysRS and disease free survival in lung cancer

**Figure 2:**
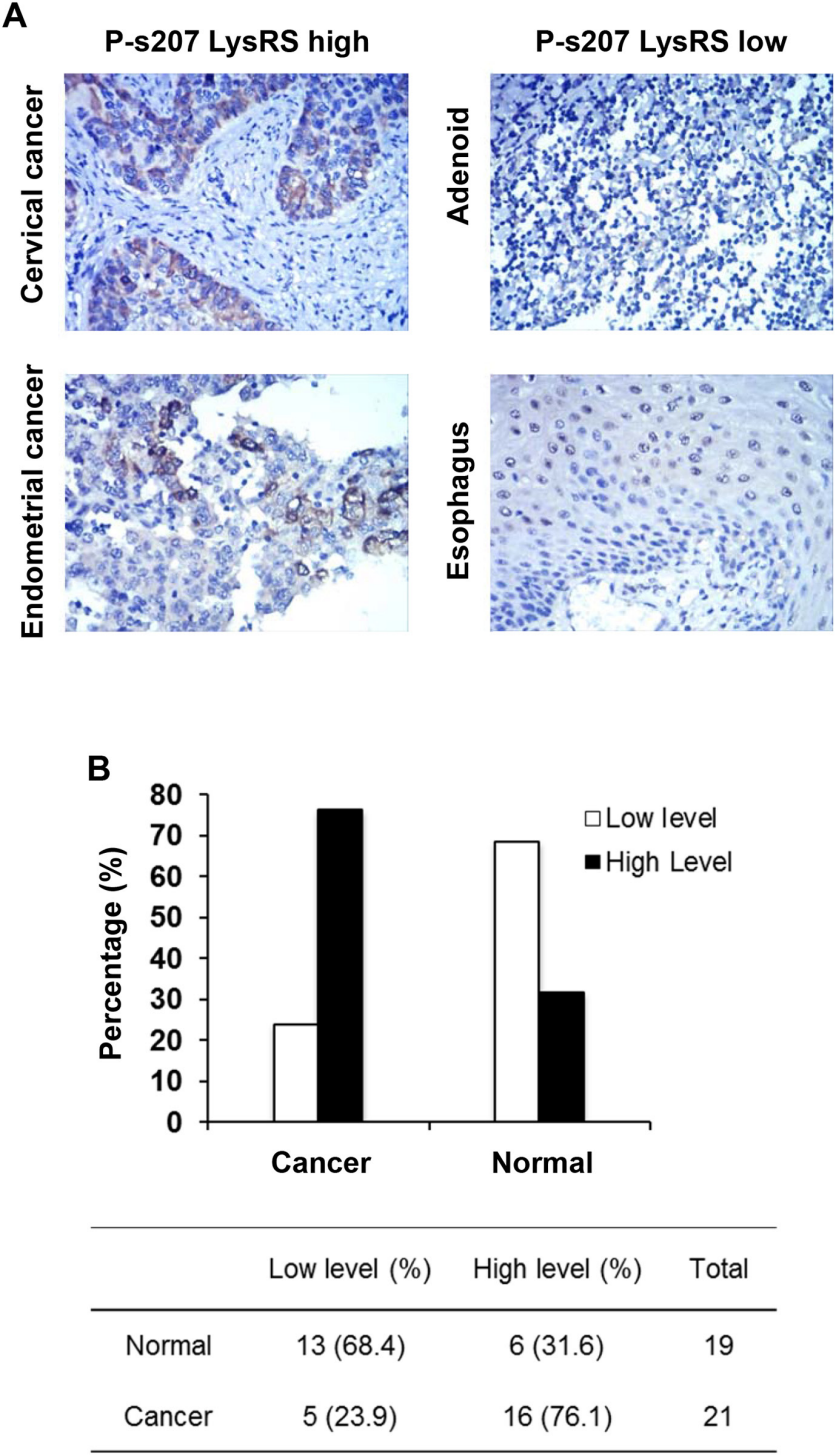
P-s207 LysRS levels in different tumor and normal tissue samples **(A)** Examples taken from the original immunohistochemistry (IHC) slides showing high and low staining levels of P-s207 LysRS. **(B)** Summary of the IHC study showing in percentage the staining of P-s207 LysRS in tumors and normal tissue.

In order to determine whether there is any association between EGFR-P-s207 LysRS and the clinical samples, a large IHC study was performed, in which 19 normal and 21 cancerous tissues from different organs with duplicates were stained for P-s207 LysRS (Figure 2A). Significantly higher staining levels of P-s207 LysRS were observed in both the cytoplasm and the nuclei of cancerous tissues compared to normal tissues (p < 0.0001) (Figure 2B).

**Figure 3:**
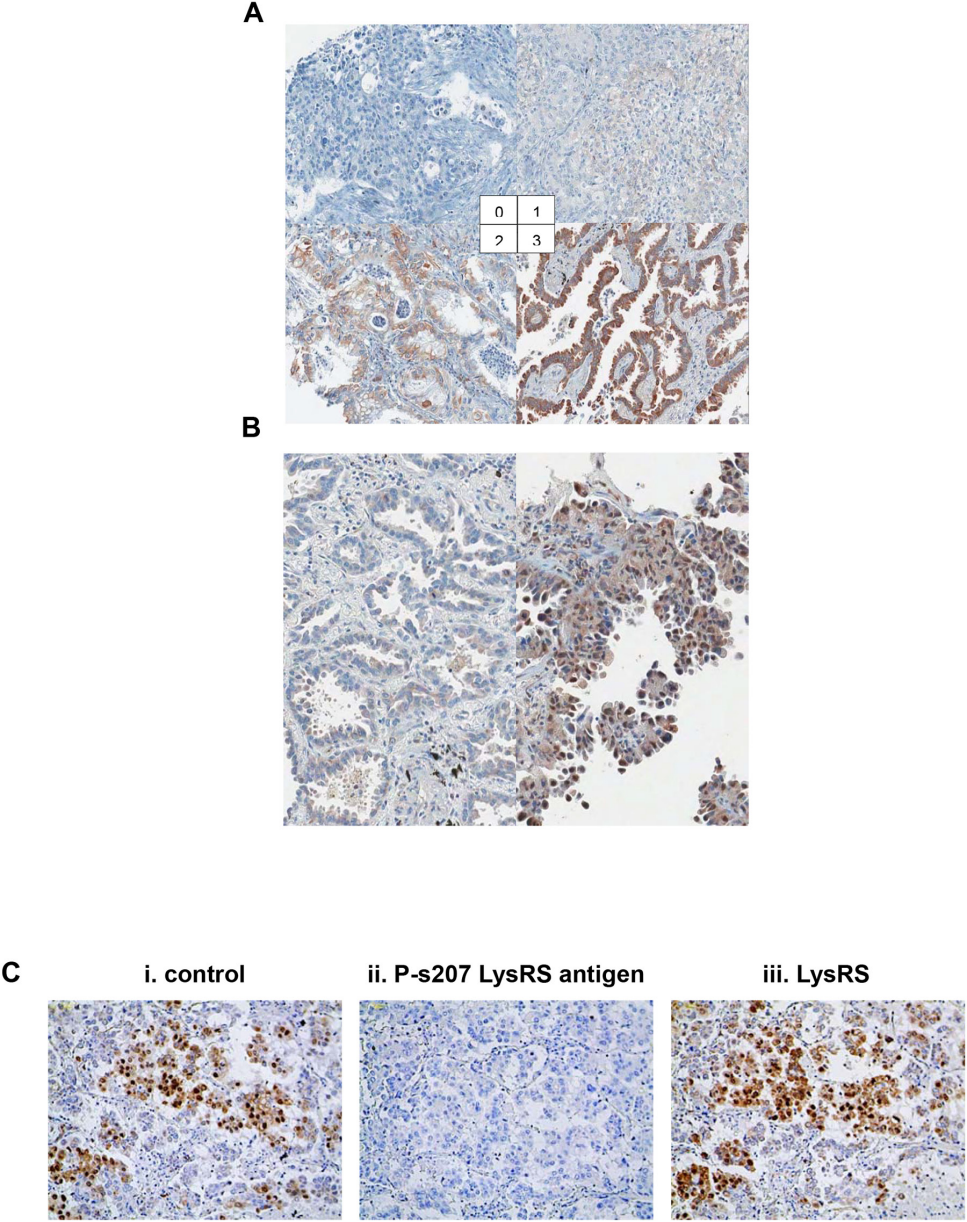
P-s207 LysRS levels in NSCLC tissues **(A)** Scoring system for cytoplasmic staining of P-s207 LysRS in NSCLC tissues. The levels of cytoplasmic P-s207 LysRS staining were classified into 4 groups (0; none or positive in less than 10% of tumor cells, 1; weak positive in more than 10% of tumor cells 2; moderate positive in more than 10% of tumor cells 3; strong positive in more than 10% of tumor cells). **(B)** Scoring for nuclear P-s207 LysRS staining in NSCLC tissues, showing no staining (left figure) vs. high staining (right figure). Cases were deemed positive for nuclear P-s207 LysRS if more than 5% of tumor cells show nuclear staining of any intensity. **(C)** Validation of P-s207 LysRS antibody in IHC staining. To validate P-s207 LysRS-specific signal in IHC, P-s207 LysRS and LysRS antigens (that were used to produce the antibodies) were pre-incubated with the antibodies. (i) Control staining with the P-s207 LysRS antibody, (ii) P-s207 LysRS staining after pre-incubated with P-s207 LysRS antigen, (iii) P-s207 LysRS staining after pre-incubation with the LysRS antigen.

Next, a large retrospective study using 242 tissues microarray samples of primary tumors from patients with non-metastatic NSCLC was conducted to investigate if P-s207 LysRS could serve as a prognostic marker (Supplementary Table 1). An example of cytoplasmic and nuclear P-s207 LysRS staining is shown in Figure 3A and 3B, respectively. The staining signal of P-s207 LysRS was blocked by P-s207 LysRS peptide, but not when using wild type LysRS peptide (Figure 3C). When all patients were considered as a single group, nuclear P-s207 LysRS was more frequently expressed in small size tumors (T1) (46.2%) compared to large size tumors (T3) (0%) (p = 0.003, n = 242) (Supplementary Table 1). In addition, higher levels of P-s207 LysRS were noted in patients without lymph node metastasis and in early stage tumors, but these findings did not reach statistical significance.

When the patients were grouped based on their EGFR status, mutated or non-mutated EGFR, it was observed that the expression of nuclear P-s207 LysRS was increased in patients harboring an EGFR mutation who had small tumors (T1) (55.3%), compared to those with larger tumors (T3) (0%) (p = 0.009, n = 140) (Supplementary Table 2). Patients with EGFR mutations without lymph node metastases also had higher nuclear expression of P-s207 LysRS (50%) as compared to patients with lymph node metastases (27.8%) (p = 0.021). In this group, stage I patients had much higher nuclear expression of P-s207 LysRS compared to stage III, but this was statistically insignificant.

**Figure 4:**
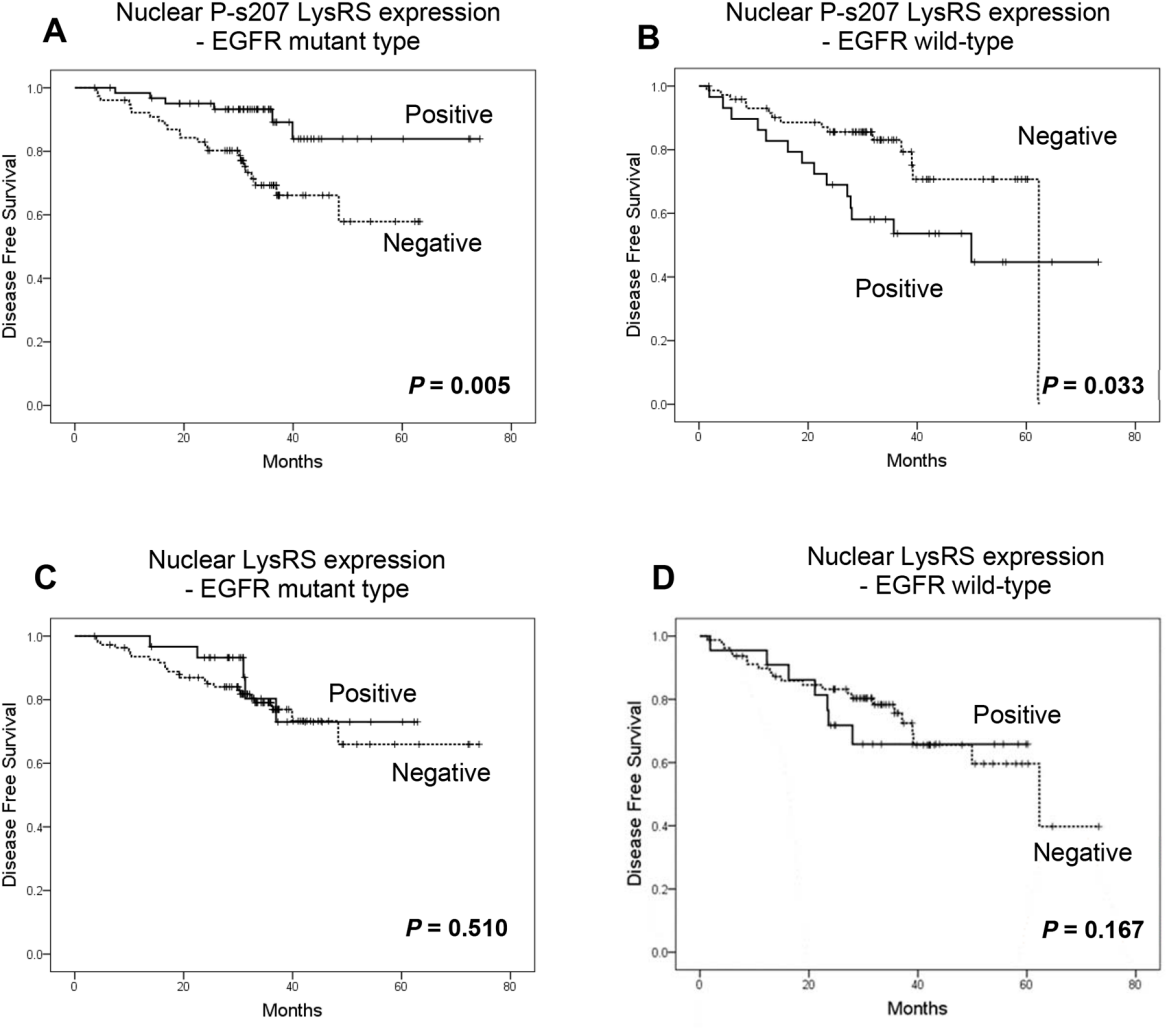
Nuclear P-s207 LysRS level is significantly associated with longer disease free survival (DFS) in NSCLC patients with mutated EGFR Disease free survival of wild-type **(A)** and mutated EGFR patients **(B)** with (positive) or without (negative) nuclear P-s207 LysRS. Disease free survival of wild-type **(C)** and mutated EGFR patients **(D)** with (positive) or without (negative) nuclear LysRS.

Having demonstrated the relationship between early NSCLC and nuclear P-s207 LysRS (especially in the EGFR mutated group), we proceeded to examine the latter’s correlation with improved disease-free survival (DFS) using Kaplan-Meier plots for NSCLC patients with wild type or mutated EGFR (Figure 4A-4D).

**Figure 5:**
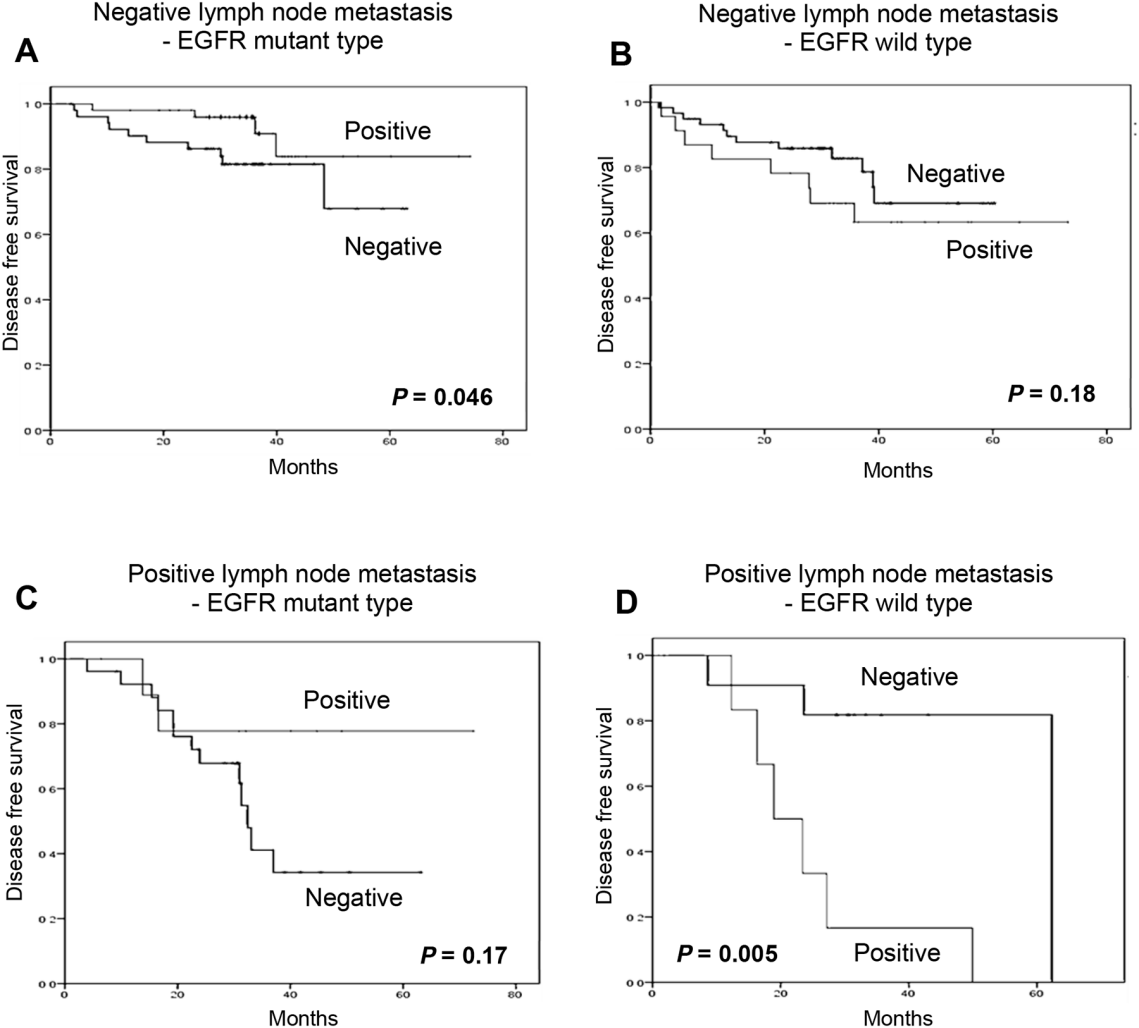
Cytosolic and nuclear LysRS levels are not associated with longer disease free survival (DFS) in NSCLC patients with mutated EGFR Disease free survival of wild-type EGFR patients with (positive) or without (negative) cytosolic **(A)** and nuclear **(B)** LysRS. Disease free survival of mutated EGFR patients with (positive) or without (negative) cytosolic **(C)** and nuclear **(D)** LysRS.

Patients who underwent surgery for their primary lung cancer and were found to harbor an EGFR mutation (mainly in exon 19 and/or 21), had a clear benefit in terms of DFS if their tumors possessed nuclear P-s207 LysRS, in contrast to those without nuclear P-s207 LysRS, with a mean DFS of 66.9 months vs. 48 months respectively (p = 0.005) (Figure 4D). Conversely, patients whose primary tumors were classified as wild type EGFR had a shorter DFS when P-s207 LysRS was present in the nucleus as compared to those without nuclear P-s207 LysRS, with a mean DFS of 45.7 months vs. 52.6 months respectively (p = 0.033) (Figure 4B). This advantage in DFS was not seen in patients with high nuclear LysRS levels regardless of their EGFR status (Figure 5).

### Nuclear P-s207 LysRS and lymph node status in lung cancer

It was also observed that patients with an EGFR mutation in exon 19 or 21 and no lymph node metastasis had longer DFS when P-s207 LysRS was present in the nucleus compared to patients with absent nuclear P-s207 LysRS, 67.5 months vs. 52.4 months respectively (p = 0.046) (Figure 5A). Conversely, EGFR wild type patients with the same lymph node status showed shorter DFS when P-s207 LysRS was present in the nucleus (Figure 5B), but this result did not reach statistical significance (p = 0.18).

The same benefit in terms of DFS was also observed in patients with lymph node metastases, with a more significant difference in DFS. In this group, EGFR mutated patients who were positive for nuclear P-s207 LysRS had a DFS of 59.7 months vs. 38 months for patients without nuclear P-s207 LysRS (p = 0.17) (Figure 5C). On the other hand, the presence of nuclear P-s207 LysRS in EGFR wild type patients predicted shorter DFS compared to patients with absent nuclear P-s207 LysRS (Figure 5D), 24.6 months vs. 53.9 months respectively, which was statistically significant (p = 0.005).

### P-s207 LysRS and colony formation in lung cancer

**Figure 6:**
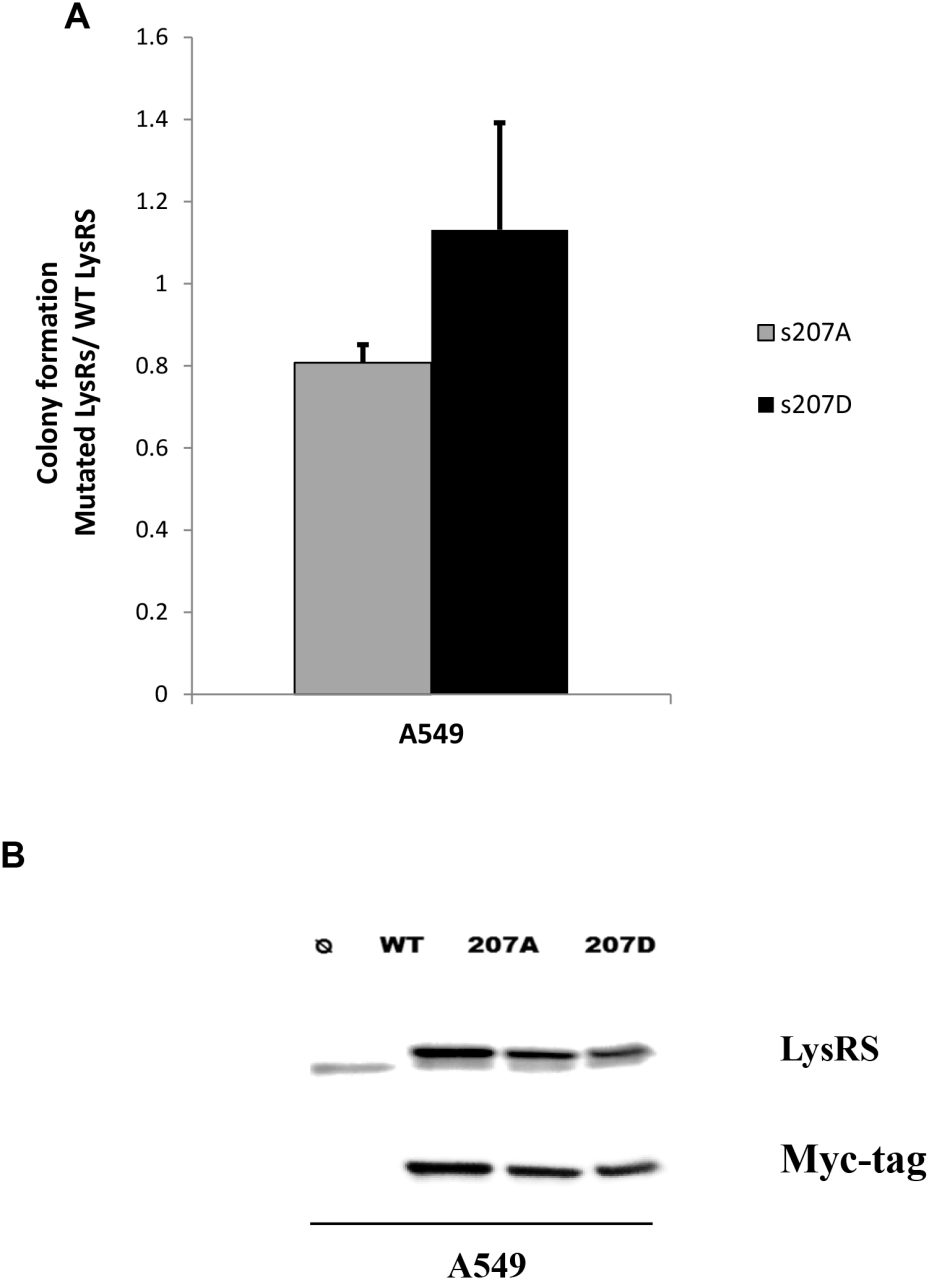
P-s207 LysRS level induces tumorigenicity **(A)** Cells were transfected with WT LysRS, s207A and s207D LysRS mutant before colony formation assay. Cells were seeded on soft agar for 8 days then fluorescent absorption was detected at 485/520 nm wavelength. Results were analyzed by T-test. Table represents 6 independent experiments. **(B)** Transfectant expression level was determined by western blotting. Error bars represent the mean ± SEM. *P < 0.05; **P < 0.01.

To evaluate the role played by P-s207 LysRS in colony formation, cells were transfected with LysRS variants prior to seeding into soft agar colony assay (Figure 6A). The variants were WT LysRS, (similar to endogenous LysRS), s207A LysRS (similar to unphosphorylated LysRS), and s207D LysRS (similar to constitutively active LysRS. A significant reduction in colony formation was observed in cells transfected with s207A-LysRS as compared to control cells transfected with WT LysRS (P <0.01) (Figure 6A), whereas s207D LysRS transfection markedly increased colonies formation (Figure 6A). The verification of the transfection procedure was observed by detecting LysRS and myc-tag levels. Similar over-expression levels of each mutant LysRS were observed. (Figure 6B). No differences were observed between the s207A and s207D in the EGFR mutated HCC827 cell line. This could be explained by the involvement of other signaling pathways such as the PI3K and STAT3 in this EGFR mutated cells [17, 18] that trigger their proliferation regardless of the phosphorylation of LyRS.

## DISCUSSION

Lung cancer is the leading cause of cancer-related mortality worldwide. Although significant progress has been achieved in lung cancer management, including diagnostic approaches, biomarkers and treatments, lung cancer is still difficult to diagnose in early stages, leading to a 17% 5-year overall survival rate according to the latest cancer statistics [19].

Studying aberrant activation of signal transduction pathways in lung cancer is important in order to better understand basic processes in this type of cancer and reveal new important therapeutic targets. In lung adenocarcinoma, mutations and over expression in the EGFR gene leads to activation of the EGFR-MAPK pathway, which may influence cell proliferation, migration and survival [20]. The EGFR pathway plays an eminent role in NSCLC, and TKIs targeting the EGFR have been shown to improve survival in metastatic patients harboring an EGFR mutation in exon 19 [21]. It has also been demonstrated that patients with metastatic lung cancer and an EGFR mutation (in exon 19 or 21) respond better to EGFR TKIs than to chemotherapy [22].

tRNA synthetases belong to a family of proteins for which multiple non translational roles, such as signal transduction, have been described. Thus, it is not surprising that some of their noncanonical functions have been reported to be critical in cancer biology [23].

We have recently described the LysRS-Ap4A signaling pathway in mast cells, in which the LysRS undergoes phosphorylation at position serine 207 following cellular activation in a MAPK dependent manner [24]. Here, we present novel evidence that phosphorylation of LysRS at position Serine 207, its consequent release from the Multi-Synthetase-Complex (MSC) and translocation to the nucleus, are not limited to mast cells and cardiomyocytes, but also occur in NSCLC.

**Figure 7:**
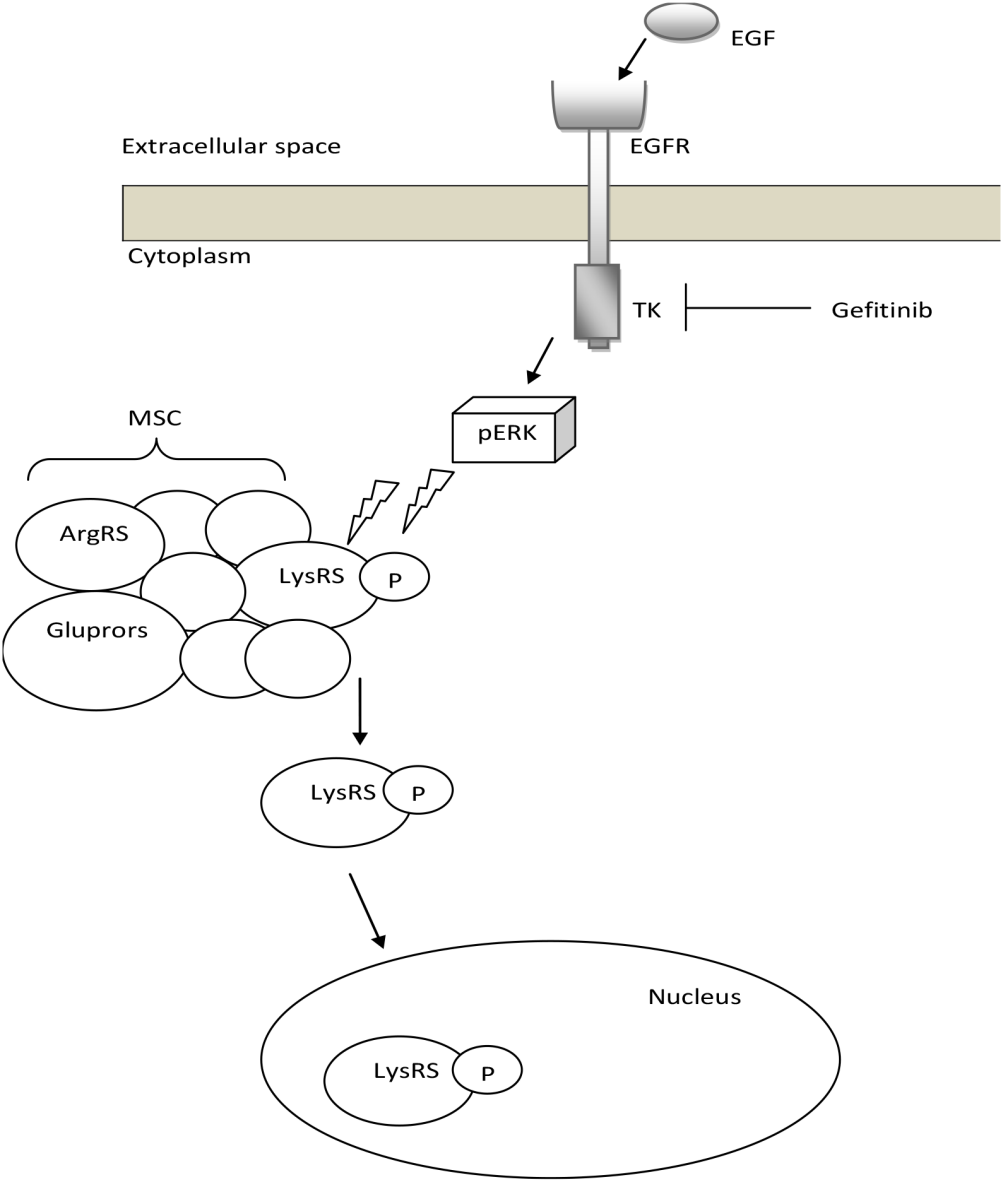
Proposed model for the EGFR-LysRS signaling pathway in lung cancer Following EGFR activation upon EGF binding, ERK is phosphorylated and activated. This results in the specific phosphorylation of LysRS at position Serine 207, its release from the MSC and translocation to the nucleus.

We demonstrate in our new *in-vitro* data that upon EGFR activation LysRS is released from the MSC in NSCLC, phosphorylated at position serine 207 in a MAPK-ERK1/2 dependent manner and translocates to the nucleus in lung cancer cells (Figure 1A and 1C, Figure 7). To confirm this finding, we demonstrated the opposite result by inhibiting the MAPK-ERK1/2 pathway in the same lung cancer cells (Figure 1D). Interestingly in lung cancer cells which harbor an activating mutation in EGFR (HCC827) we observed that high percentage of the LysRS are phosphorylated at position s207 (Figure 1D), implying that at least *in vitro*, aberrant activation of EGFR can lead to over-activation of the LysRS pathway.

There is a great interest in the role of EGFR TKIs as agents for use in adjuvant treatments for patients post-surgery. The need for improved therapies for these patients is accentuated by recent data demonstrating significantly shorter DFS of EGFR mutated stage 3 lung cancer patients compared to wild type patients [25]. Indeed, a large clinical trial focusing on adjuvant therapies for lung cancer has begun in the USA, which includes data mining for biologic factors which may influence survival [26].

However, to date, there is no proof that adjuvant treatment with an EGFR inhibitor can prolong survival, even for EGFR mutated tumors [27]. In addition, the duration of response to such treatments and the development of resistance are unpredictable [28]. Therefore, it is simply insufficient to target a single pathway independently and a deeper understanding of cancer behavior is required.

Our study used tissues obtained from mostly early stage lung cancer patients from Korea, where there is a high percentage of EGFR mutation positive lung cancer. Based on our large, retrospective IHC study of NSCLC patients, we demonstrate that in the subgroup of patients with an EGFR mutation, there is significantly longer DFS if nuclear P-s207 LysRS is detected. This result is interesting since it implies that not all EGFR mutated cancers behave similarly, and that activation of the LysRS pathway only occurs in some cases.

The data were analyzed in accordance to different variables and did not show any significant correlation between P-s207 LysRS and survival. However we did note that in patients with wild type EGFR and lymph node metastases, P-s207 LysRS nuclear expression appear to be associated with worse prognosis. Since the numbers are relatively small, this is merely a hypothesis that will require further validation in a much larger cohort.

A possible explanation for this observation could be that in EGFR wild type NSCLC, signaling pathways other than the MAPK/ERK pathway, such as the signal transducer and activator of transcription 3 (STAT3) pathway [17] or the phosphoinositide 3-kinase (PI3K) pathway [18], play a central role. For example, approximately one-third of NSCLC patients with KRAS EGFR mutations harbor mutations in serine/threonine kinase 11 (STK11), which skews their response to MEK inhibitors [29]. Recently in a large series of NSCLC patients, Inoue and colleagues revealed two new markers for NSCLC CD73 and A2A adenosine receptor [30]. These two markers are highly expressed in EGFR mutated patients. The mode of action of these markers are related to immune activation and are very different from the mode of action of which P-s207 LysRS is related to signal transduction mechanisms. It would be of great interest to try and combine the use of these different markers to obtain a better prognostic marker in EGFR wild type and mutated patients in future studies.

The mechanisms underlying the converse result observed in non-mutated EGFR cells are unclear, and could be attributed to the differential signal transduction networks induced in these cells. Nonetheless, the results are of great clinical potential and could be an additional prognostic tool for oncologists in the future in determining recurrence probability more accurately in NSCLC patients after primary surgery and for those requiring adjuvant chemotherapy.

## MATERIALS AND METHODS

### Cell culture and reagents

Three lung cancer cell lines, A549 (a human adenocarcinoma cell line with wild type EGFR), HCC827 (a human adenocarcinoma cell line harboring an exon 19 deletion in the EGFR), and H460 (a human large cell carcinoma), were obtained from American Type Culture Collection (Manassas, USA). All cell lines were cultured and maintained in RPMI 1640 medium (HyClone, Logan, USA) containing 10 % FBS. EGF and Gefitinib were purchased from Sigma-Aldrich (St. Louis, USA).

### Western blot analysis

Cultured cells were harvested and lysed on ice with lysis buffer, containing 25 mM Tris-HCl (pH 7.4), 150 mM NaCl, 0.5 mM EDTA, 0.5% Triton X-100, 10 mM NaF, 1 mM sodium orthovanadate, 10% glycerol and protease inhibitors (Calbiochem, San Diego, USA), for western blot. Protein concentration was determined by Bradford assay (Bio-Rad, Hercules, USA). Proteins were resolved by 10 % SDS-PAGE gels and transferred to nitrocellulose membranes. Visualization of the proteins was performed by chemiluminescence (AGFA, CP1000, Mortsel, Belgium). The anti-LysRS antibody was purchased from Neomics (Seoul, Korea). The anti-phosphorylated-LysRS antibody was custom-made against s207 phosphorylation (Abmart Laboratories, China). The anti-EGFR antibody was purchased from Santa Cruz Biotechnology (Dallas, USA), the anti-GluProRS and GlyRS antibodies from Abcam (Cambridge, UK), anti-ERK1/2 and anti-pERK1/2 antibodies from Cell Signaling Technology (Danvers, USA).

### Immunodepletion assay

Cells were starved for 2 hr in serum-free medium and lysed on ice after treatment. To deplete the MSC, anti-GluProRS and anti-GlyRS (separate samples, control) antibodies were added for 4hr at 4°C and then depleted twice by using protein G agarose beads (Invitrogen, Carlsbad, USA). The anti-GlyRS and GluProRS antibodies were purchased from Abcam (Cambridge, UK, ab42905, ab31531). These antibodies showed target specificity and were validated in references [31–35]. In western blotting, they showed single band, predicted exact band size, and were validated in 2 and 3 references. Western blots for LysRS and P-s207 LysRS were performed using the supernatant and pellet.

### Cell fractionation

Cells from each 10 cm plate were homogenized in 1 ml of fractionation buffer, containing 25 mM Tris-HCl (pH 7.4), 1 mM EDTA, 0.5 mM EGTA, 10 mM NaCl, 0.1 % NP-40, 10 mM NaF, 1 mM sodium orthovanadate and protease inhibitors, by a Dounce homogenizer (Sigma-Aldrich). A one-tenth volume of fractionation buffer with 2.5 M sucrose was added to the homogenate. The homogenate was centrifuged at 1,000 g for 10 min. The supernatant was designated as cytosolic fraction. The pellet was resuspended in fractionation buffer with 50% sucrose and centrifuged at 10,000 g for 5 min. The pellet was used as nuclear fraction.

### ELISA competitive assay

96 well immunoplates (Corning, New York, USA) were coated with 100 nM of P-s207 LysRS peptide (Ag) and blocked with 3% skim milk. Anti-P-s207 LysRS antibody (10 nM) was pre-incubated with different concentrations of either P-s207 LysRS Ag or LysRS Ag for 2 hr at 20°C. The pre-incubated complex was applied to the coated plate for 30 min at RT. The antibodies, which bound to plate-coated P-s207 LysRS, were detected with anti-rabbit-HRP antibody (Thermo Scientific, Lafayette, USA). Signals were developed with TMB solution (Sigma-Aldrich, St. Louis, USA) and read with the Synergy microplate reader (Biotek, Windooski, USA).

### Patients and samples

Tissue micro-array (TMA) samples were obtained from 242 patients with mostly adenocarcinoma lung cancer (96.7%), who underwent surgery to remove their primary tumor at the Department of Thoracic Surgery of the Seoul National University Hospital. The patient samples in this study were taken from individuals with stages I, II or III lung cancer and were divided into two main groups – wild type EGFR and EGFR mutations (exon 19 deletion, and exon 21 mutation). All samples were stained and reviewed at the Pathology Department of the Seoul National University Hospital. This study was approved by the Institutional Review Board of Seoul National University Hospital (IRB No. 1508-060-694). Informed consent for participation in the study was waivered by the Institutional Review Board of Seoul National University Hospital on the basis that this study was a retrospective study using archived material, and did not increase risk to the patients.

### Immunohistochemistry (IHC)

TMAs were automatically stained using automated immune-stainer, Ventana Benchmark XT (Roche, USA). OptiView and ultraView kits (Ventana, Tuscon, USA) were used for LysRS and P-s207 LysRS staining, respectively. Briefly, TMAs were pre-incubated with Cell Conditioning solution 1 (CC1) for 8 min. The diluted primary antibodies were incubated with the TMAs for 16 min. Stained TMAs were scanned and analyzed by Aperio ScanScope (Aperio Technologies, Vista, USA). Methodologically, the quantitation of positive cells were automatically performed using Aperio ScanScope software after virtual microscope scanning following the tuning of the software by the pathologist in terms of intensity and tumor/nuclear size. The detailed cut off the values used are as follows: For the Cytoplasmic LysRS and P-s207 LysRS staining, 0: none or positive in less than 10% of tumor cells, 1+: weak positive in more than 10% of tumor cells, 2+: moderate positive in more than 10% of tumor cells, 3+: strong positive in more than 10% of tumor cells. Cases with score 2+ or 3+ were deemed positive for LysRS and P-s207 LysRS cytoplasmic expression. Cases were deemed positive for nuclear LysRS if more than 10% of tumor cells show nuclear staining in any intensity (1+ or more) and positive for nuclear P-s207 LysRS if more than 5% of tumor cells show nuclear staining in any intensity (1+ or more).

### Neutralizing IHC

Each tissue slide was deparaffinized with xylene and rehydrated in different percentages of ethanol (100, 95, 70 and 50%) for 3 min each. Endogenous peroxidases were blocked with 3% H2O2 (Sigma-Aldrich, St. Louis, USA) in methanol for 10 min. The slides were rinsed in PBS twice for 5 min after each step. Antigen retrieval was performed using 10 mM citric buffer (pH 6.0) at 95°C for 10 min. Anti-P-s207-LysRS antibody, diluted in 0.5% BSA in PBS, was pre-incubated either with 1μM P-s207-LysRS or 1μM LysRS peptide antigen (Ag) (EITLLS(p)PCLHM, EITLLSPCLHM, respectively, for 2 hr at 20°C. After cooling down the slides, the tissue samples were blocked with 4% BSA in PBS for 1 hr at room temperature (RT), followed by application of the pre-incubated antibody-antigen solution for 1 hr. Anti-rabbit-HRP (Dako, Carpinteria, USA) was then applied for 1 hr. P-s207 LysRS was detected with DAB and Chromogen mixture (Dako, Carpinteria, USA). After washing with PBS, a nuclear counter staining was carried out with Mayer’s haematoxylin (Sigma-Aldrich, St. Louis, USA). The tissue slides were dehydrated in 95% and 100% ethanol, twice for 5 min each, cleared in xylene and then mounted.

### Plasmid construction

Human LysRS was subcloned into the EcoRI and XbaI sites of the pSC2+MT vector (Invitrogen). This vector was used for the production LysRS mutant by site- directed mutagenesis in which serine was replaced by alanine at 207 (LysRS s207A). Human LysRS s207A variant was subcloned into pCMV/myc/cyto and pCMV/myc/nuc vectors. The fidelity of all constructs was verified by direct sequencing.

### Transfection reagents and procedure

Cells were seeded at a density of 2 × 10^5^ cells in each well of a 6-well culture plate. 24 hours after seeding, the cells were transfected and incubated for 24 h. The procedure of jetPRIME (Polyplus transfection, Illkirch, France) mediated transfection in 6-well culture plate was as follows. Plasmid DNA (3 μg) and 9 μl jetPRIME reagent were diluted into 200 μl buffer, and incubated for 10 min at room temperature before being added to each well.

### Colony formation assay

A total of 1 × 10^3^ cells were seeded in each well of a 96-well plate underlying a base of soft agar layer as mediated in CytoSelect™ 96-Well Cell Transformation Assay (Soft Agar Colony Formation) protocol. Cells were allowed to grow for 8 days before being solubilized, lysed and detected by the CyQuant GR Dye in a fluorescence plate reader using a 485/520 nm filter set.

### Statistical analysis

Statistical analyses were performed using SPSS software (version 21; IBM Corp., New York, NY, USA). Comparisons between variables were performed using the χ2 test or Fisher’s exact test. A tissue micro array database of lung cancer specimens obtained upon resection (n = 242) was used to analyze the effect of P-s207 LysRS on Disease Free Survival (DFS). Survival analysis was performed using the Kaplan-Meier method with the log-rank test. For all statistical analyses, two-sided P values < 0.05 were considered to be statistically significant. Reagents and additional methods, including molecular and cellular assays, were performed as described in *SI Materials and Methods*.

## SUPPLEMENTARY MATERIALS FIGURE AND TABLES


