# Correction: Serine 207 phosphorylated lysyl-tRNA synthetase predicts disease-free survival of non-small-cell lung carcinoma

**DOI:** 10.18632/oncotarget.26387

**Published:** 2018-11-16

**Authors:** Suliman Boulos, Min Chul Park, Marian Zeibak, Shen Yun Foo, Yoon Kyung Jeon, Young Tae Kim, Alex Motzik, Sagi Tshori, Tamar Hamburger, Sunghoon Kim, Hovav Nechushtan, Ehud Razin

**Affiliations:** ^1^ Department of Oncology, Hadassah Hebrew University Hospital, Jerusalem, Israel; ^2^ Medicinal Bioconvergence Research Center, Seoul National University, Seoul, Korea; ^3^ Department of Biochemistry and Molecular Biology, Institute for Medical Research Israel-Canada, The Hebrew University of Jerusalem, Jerusalem, Israel; ^4^ NUS-HUJ-CREATE Cellular & Molecular Mechanisms of Inflammation Program, Department of Microbiology and Immunology, National University of Singapore, Singapore; ^5^ Department of Pathology, Seoul National University Hospital, Seoul, Korea; ^6^ Department of Thoracic and Cardiovascular Surgery, Seoul National University Hospital, Seoul, Korea; ^7^ Kaplan Medical Center, Rehovot, Israel; ^8^ Sharett Institute of Oncology, Hadassah Hebrew University Hospital, Jerusalem, Israel

**This article has been corrected:** The correct title for figure 5 is given below:

**Figure 5: Subgroup analysis of nuclear P207 LysRS according to Lymph nodes positivity.** Disease free survival of wild-type EGFR patients with (positive) or without (negative) cytosolic **(A)** and nuclear **(B)** LysRS. Disease free survival of mutated EGFR patients with (positive) or without (negative) cytosolic **(C)** and nuclear **(D)** LysRS.

Original article: Oncotarget. 2017; 8:65186-65198. https://doi.org/10.18632/oncotarget.18053

