# Supplementary material for: Serine 207 phosphorylated lysyl-tRNA synthetase predicts disease-free survival of non-small-cell lung carcinoma
Source: Oncotarget. 2017 May 22;8(39):65186–98. doi: 10.18632/oncotarget.18053 (PMC5630322; doi:10.18632/oncotarget.18053)
Supplement: Supplementary file 1 [file oncotarget-08-65186-s001.pdf]

Serine 207 phosphorylated lysyl-tRNA synthetase predicts disease-free survival of non-small-cell lung carcinoma

SUPPLEMENTARY MATERIALS

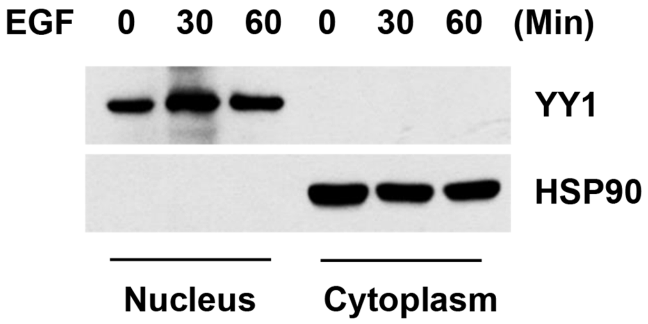

Supplementary Figure 1: Intracellular localization of free LysRS and P-s207 LysRS, A549 cells were starved for 2 hr, followed by incubation with EGF, and LysRS levels were determined in nuclear and cytoplasmic fractionations by immunoblotting as was shown in Figure 1C. Ying-Yang protein1 (YY1) (a transcriptional repressor found only in the nucleus) and heat shock protein 90 (HSP90) (a chaperone protein found only in the cytoplasm) were used as controls.

Supplementary Table 1: Expression of LysRS and P-s207 LysRS according to pathological characteristics

Pathologic characteristics	Cytoplasmic LysRS (%)			Nuclear LysRS (%)			Cytoplasmic P-s207 LysRS (%)			Nuclear P-s207 LysRS (%)		
	**High**	**Low**	***P***	**High**	**Low**	***P***	**High**	**Low**	***P***	**High**	**Low**	***P***
Tumor size												
T1	23.8	76.2	>0.05	21.5	78.5	>0.05	37.7	62.3	>0.05	46.2	53.8	0.003
T2	22.5	79.4		20.6	79.4		31.4	68.6		31.4	68.6	
T3	10	90		30	70		10	90		0	100	
Lymph node metastasis												
Absent	19.3	80.7	0.017	18.2	81.8	0.021	33.7	66.3	>0.05	40.1	59.9	>0.05
Present	34.5	65.5		32.7	67.3		34.5	65.5		30.9	69.1	
P stage												
I	18.5	81.5	0.038	17.3	82.7	0.045	35.7	64.3	>0.05	41.7	58.3	>0.05
II	28.2	71.8		28.2	71.8		25.6	74.4		25.6	74.4	
III	37.1	62.9		34.3	65.7		34.3	65.7		34.3	65.7	

Expression of LysRS and P-s207 LysRS in the cytoplasm and nucleus of non-small cell lung cancer according to tumor size (T), lymph node involvement, and stage.

Supplementary Table 2: Expression of LysRS and P-s207 LysRS according to EGFR status and pathological characteristics

Pathological characteristics	EGFR wild type						EGFR mutant type					
	Cytoplasmic P-s207 LysRS (%)			Nuclear P-s207 LysRS (%)			Cytoplasmic P-s207 LysRS (%)			Nuclear P-s207 LysRS (%)		
	High	Low	*P*	High	Low	*P*	High	Low	*P*	High	Low	*P*
Tumor size												
T1	31.5	68.5	>0.05	33.3	66.7	>0.05	42.1	57.9	>0.05	55.3	44.7	0.009
T2	34.1	65.9		29.3	70.7		29.5	70.5		32.8	67.2	
T3	0	100		0	100		33.3	66.7		0	100	
Lymph node invasion												
Absent	28.9	71.1	>0.05	22.7	72.3	>0.05	37.5	62.5	>0.05	50	50	0.021
Present	36.8	63.2		36.8	63.2		33.3	66.7		27.8	72.2	
P stage												
I	31	69	>0.05	29.6	70.4	>0.05	39.5	60.8	>0.05	50.5	49.5	>0.05
II	22.2	77.8		22.2	77.8		28.6	71.4		28.6	71.4	
III	38.5	61.5		38.5	61.5		31.8	68.2		31.8	68.2	
